# Provider‐Level Variation in Smoking Cessation Assistance Provided in the Cardiology Clinics: Insights From the NCDR PINNACLE Registry

**DOI:** 10.1161/JAHA.118.011307

**Published:** 2019-06-28

**Authors:** Mayank Sardana, Yuanyuan Tang, Jared W. Magnani, Ira S. Ockene, Jeroan J. Allison, Suzanne V. Arnold, Phillip G. Jones, Thomas M. Maddox, Salim S. Virani, David D. McManus

**Affiliations:** ^1^ University of California San Francisco San Francisco CA; ^2^ Mid America Heart Institute Kansas City KS; ^3^ University of Pittsburgh School of Medicine Pittsburgh PA; ^4^ University of Massachusetts Medical School Worcester MA; ^5^ Washington University School of Medicine St Louis MO; ^6^ Michael E. DeBakey Veterans Affairs Medical Center Section of Cardiovascular Research Department of Medicine Baylor College of Medicine Houston TX

**Keywords:** primary prevention, quality of care, registry, smoking, Quality and Outcomes, Primary Prevention, Risk Factors

## Abstract

**Background:**

Studies show suboptimal provision of smoking cessation assistance (counseling or pharmacotherapy) for current smokers attempting to quit. We aimed to identify smoking cessation assistance patterns in US cardiology practices.

**Methods and Results:**

Among 328 749 current smokers seen between January 1, 2013, and March 31, 2016, in 348 NCDR (National Cardiovascular Data Registry) PINNACLE (Practice Innovation and Clinical Excellence)‐affiliated cardiology practices, we measured the rates of cessation assistance. We used multivariable hierarchical logistic regression models to determine provider‐, practice‐, and patient‐level predictors of cessation assistance. We measured provider variation in cessation assistance using median rate ratio (the likelihood that the same patient would receive the same assistance at by any given provider; >1.2 suggests significant variation). Smoking cessation assistance was documented in only 34% of encounters. Despite adjustment of provider, practice, and patient characteristics, there was large provider‐level variation in cessation assistance (median rate ratio, 6 [95% CI, 5.76–6.32]). Practice location in the South region (odds ratio [OR], 0.48 [0.37–0.63] versus West region) and rural or suburban location (OR, 0.92 [0.88–0.95] for rural; OR, 0.94 [0.91–0.97] for suburban versus urban) were associated with lower rates of cessation assistance. Similarly, older age (OR, 0.88 [0.88–0.89] per 10‐year increase), diabetes mellitus (OR, 0.84 [0.82–0.87]), and atrial fibrillation (OR, 0.93 [0.91–0.96]) were associated with lower odds of receiving cessation assistance.

**Conclusions:**

In a large contemporary US registry, only 1 in 3 smokers presenting for a cardiology visit received smoking cessation assistance. Our findings suggest the presence of a large deficit and largely idiosyncratic provider‐level variation in the provision of smoking cessation assistance.

**Clinical Perspective**

**What Is New?**

- Of all current smokers who were seen in the outpatient cardiology practices enrolled in the PINNACLE (Practice Innovation and Clinical Excellence) registry, only 1 in 3 received smoking cessation assistance.
- Despite adjusting for various predictors, a large provider‐level variation in smoking cessation assistance persisted.

**What Are the Clinical Implications?**

- Our findings provide real‐world data on the large deficit and largely idiosyncratic provider‐level variation in the smoking cessation assistance practices.
- These findings call for immediate action from providers and public health organizations to improve the adherence to the provision of smoking cessation assistance in outpatient cardiology practices.

## Introduction

Cigarette smoking is a modifiable risk factor for cardiovascular disease (CVD), and smoking cessation leads to 2‐ to 3‐fold reduction in the risk for incident CVD and mortality within 5 years of smoking cessation.^1^, ^2^, ^3^, ^4^, ^5^ Despite the clear benefits of smoking cessation and availability of effective cessation therapies,^6^ nationally representative data from community‐based samples suggest that only 1 in 3 smokers report receiving smoking cessation assistance (counseling and/or pharmacotherapy) while attempting to quit.^5^ Patient‐survey based studies have similarly revealed a large deficit in the provision of smoking cessation assistance in outpatient clinics,^7^, ^8^, ^9^, ^10^ which matches up with the documentation of assistance in the electronic medical records.^11^ Even in clinical trial settings (which inherently bring in the bias because of better clinical practice), the assistance rates have been reported to be low.^12^

The lack of familiarity with smoking cessation guidelines, inadequate reimbursement for counseling, and physician's own smoking status have been identified in smaller single‐center studies as the provider‐level factors associated with poor adherence to smoking assistance recommendations.^13^ A recent survey of nearly 150 US‐based cardiologists conducted by the American College of Cardiology acknowledged the presence of a significant deficit in comfort of providers for referral to the smoking cessation programs and prescription of evidence‐based pharmacotherapy to assist cessation attempts.^14^

To date, no large contemporary studies have assessed national smoking cessation assistance patterns using registry‐based data, which is inherently more likely to be representative of “real‐world” practice patterns as compared with survey data. The NCDR (National Cardiovascular Data Registry's) PINNACLE (Practice Innovation and Clinical Excellence) is the largest outpatient cardiovascular registry in the world.^15^ PINNACLE continuously collects data from the outpatient cardiology visits at the participating sites using validated mapping algorithms. We performed a cross‐sectional analysis leveraging the PINNACLE data to (1) measure the rates of smoking cessation assistance provided to current smokers; (2) identify the predictors of smoking cessation assistance; and (3) measure provider‐level variation in cessation assistance after adjusting for provider, practice, and patient characteristics.

## Methods

In accordance with the NCDR policies, data, methods used in the analysis, and materials used to conduct the research will not be made available by the authors. A requisition to obtain the data can be directly submitted to NCDR for purposes of reproducing the results or replicating the procedure.

### Study Sample

The sample for our study was derived from the NCDR PINNACLE registry. A total of 348 US ambulatory cardiology practices currently voluntarily participate in the PINNACLE registry. Data are collected prospectively using a validated electronic medical record mapping algorithm that captures a variety of relevant data elements such as demographics, medical conditions, physical examination findings, medications, laboratory values, diagnostic testing, and interventions.^16^ Additionally, practice characteristics such as practice location (US Census region,^17^ rural versus suburban versus urban location), and provider type (physician versus advanced practice provider) are collected. The St Luke's Mid‐America Heart Institute (Kansas City, MO) performs periodic audits to ensure the validity and quality of collected data. Waiver of written informed consent and authorization for this study was granted by Chesapeake Research Review Incorporated.

**Figure 1:**
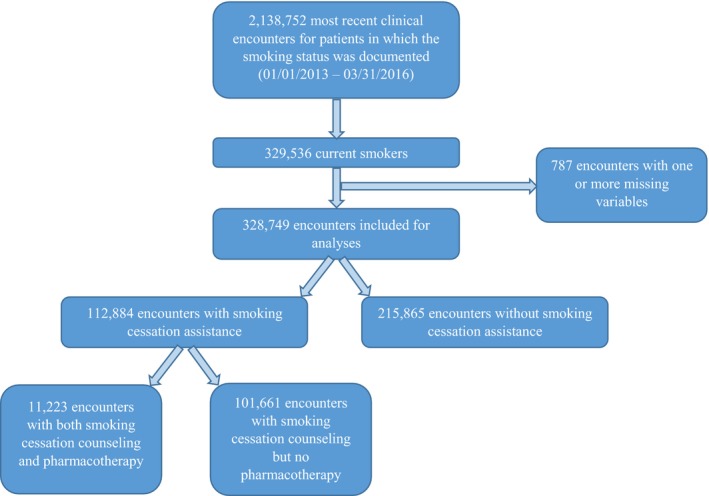
Flowchart depicting development of study sample.

Figure 1 depicts the flowchart for development of a sample for our current study. Briefly, all the electronic medical records for patients seen in US cardiology practices (irrespective of the reason for presentation) enrolled in the PINNACLE registry from January 1, 2013, to March 31, 2016, were screened using the algorithm to select the most recent encounters of patients for whom smoking cessation was assessed during the encounter *and* who were identified as “current smokers.” A total of 329 536 records were identified as the most recent encounters for current smokers. Because of 1 or more missing variables, 787 (0.24%) records were excluded. Therefore, our final sample size was 328 749 unique clinical encounter records across 348 practices participating in the PINNACLE registry.

### Outcome Variables

For purposes of our analyses, the NCDR PINNACLE registry v1.5 data collection form was used.^16^ The PINNACLE electronic medical record mapping algorithm has been validated to search for keywords pertaining to evidence‐based methods of smoking cessation counseling (eg, encourage to set a quit date, discuss medications, or refer to smoking cessation treatment).^6^ If any of the validated keywords are noted in the encounter record, it prompts a “yes” response to the “smoking cessation counseling provided” data element. Additionally, those encounters with a “yes” response to “bupropion,” “nicotine replacement therapy,” or “varenicline”^18^ data elements were identified as the ones that received pharmacotherapy (in addition to the counseling). If the response to the above data elements was “no,” we considered those encounters as the ones in which smoking cessation assistance was not documented.

### Predictor Variables

Predictor variables in our analyses included (1) provider type (physician versus advanced practice providers), (2) practice characteristics (practice region and urbanity), (3) patient demographics (age, sex), and (4) medical comorbidities (hypertension, diabetes mellitus, dyslipidemia, coronary artery disease, peripheral arterial disease, transient ischemic attack (TIA) or cerebrovascular accident, heart failure, atrial fibrillation or flutter, and prior vascular intervention). Provider type was categorized as physician versus advanced provider practitioners (physician assistants and nurse practitioners) on the basis of national provider identification enumeration.^19^ Practice region was categorized on the basis of the 4 US Census regions (Northeast, Midwest, South, and West).^17^ A significant proportion of encounters in the PINNACLE registry have the race and insurance status missing. Therefore, we decided to not include those variables in our analyses.

### Statistical Analyses

Baseline characteristics are presented as means±SD for continuous variables and as numbers and percentages for categorical variables. We utilized multilevel hierarchical logistic regression to models to measure (1) the provider‐level variation in smoking cessation assistance, and (2) the association of the predictor variables with smoking cessation assistance. To measure the magnitude of provider‐level variation, median rate ratio (MRR) was calculated. MRR estimates the probability that 2 randomly selected patients with similar characteristics (covariates) receiving care from 2 different providers in practice with similar characteristics (census region and urbanity) will receive varying treatment. By definition, MRR is always >1, as MRR of 1 would suggest no variation between providers. MRR >1.20 reflects a significant variation in practice.^20^ In the first regression model (empty model), provider number was included as a random effect variable to estimate the variation in smoking cessation assistance between providers. The second regression model included practice characteristics (census region and urbanity) and provider type (physician versus advanced practice providers) as fixed‐effect variables. The third regression model additionally included patient‐level variables (age, sex, hypertension, diabetes mellitus, dyslipidemia, coronary artery disease, peripheral arterial disease, TIA or cerebrovascular accident, heart failure, atrial fibrillation or flutter, and prior vascular intervention). No significant collinearity was identified among various predictive variables. A 2‐tailed *P*≤0.05 was considered significant. MRR was calculated with each regression model as a measure of residual provider‐level variation with inclusion of the various provider, practice, and patient characteristics. SAS version 9.4 (SAS Institute, Cary, NC) was used to perform analyses.

## Results

The baseline patient, provider, and practice characteristics of the study sample are presented in Table 1. Our sample consisted of clinical encounters of middle‐aged patients with a slight male predominance (mean age, 57±16 years; 54% male). There was a high prevalence of the cardiovascular risk factors, such as hypertension (61%), dyslipidemia (54%) and diabetes mellitus (20%). Nearly 1 in 3 patients had a diagnosis of coronary artery disease, whereas 7% had a diagnosis of TIA or ischemic stroke. The majority of patients were seen by a physician provider (93%). The majority of encounters in our sample (current smokers) were generated from the cardiology practices located in the South Census region (64%).

**Table 1:** Baseline Patient‐, Provider‐, and Practice‐Level Characteristics of Study Patients by Smoking Cessation Assistance

Variable, n (%) Unless Specified	Smoking Cessation Assistance		*P* Value
	Yes (N=112 884)	No (N=215 865)	
Patient‐level characteristics			
Age, y, mean±SD	59±14	57±17	<0.001
Women	50 666 (44%)	101 438 (47%)	<0.001
Hypertension	79 428 (70%)	119 847 (55%)	<0.001
Diabetes mellitus	23 519 (21%)	42 657 (20%)	<0.001
Dyslipidemia	73 652 (65%)	103 105 (48%)	<0.001
Coronary artery disease	51 576 (46%)	65 214 (30%)	<0.001
Peripheral arterial disease	21 614 (19%)	20 672 (10%)	<0.001
TIA or ischemic stroke	10 624 (9%)	13 099 (6%)	<0.001
Heart failure	18 866 (17%)	25 193 (12%)	<0.001
Atrial fibrillation or flutter	17 158 (15%)	26 546 (12%)	<0.001
Prior vascular intervention	18 250 (16%)	19 782 (9%)	<0.001
Provider‐ and practice‐level characteristics			
Physician provider	106 113 (94%)	201 029 (93%)	<0.001
US Census region			
Northeast region	19 073 (17%)	24 296 (11%)	<0.001
Midwest region	21 495 (19%)	24 161 (11%)	
South region	61 877 (55%)	149 828 (69%)	
West region	10 429 (9%)	17 580 (8%)	
Urbanity			
Rural	44 870 (41%)	95 853 (46%)	<0.001
Suburban	43 121 (39%)	76 592 (37%)	
Urban	22 493 (20%)	35 889 (17%)	

TIA indicates transient ischemic attack.

Of 328 749 most recent encounters of current smokers, smoking cessation assistance was documented in a third of the encounters (n=112 884, 34%; Figure 1). Assessing the rates of smoking cessation assistance using only the most recent clinical encounters could potentially underestimate the actual rates of smoking cessation assistance (eg, if the assistance was provided during another clinical encounter in close temporal proximity). Therefore, we measured the rates of smoking cessation assistance during “any” clinical encounters during the study period (January 2013 to March 2016), and the rates were not significantly different from our results using the last clinical encounters (33% versus 34%). Of 112 884 encounters in which smoking cessation assistance was documented, smoking cessation pharmacotherapy (bupropion, nicotine replacement therapy, or varenicline) was prescribed in only 10% (11 223) of the encounters (Table S1). In an unadjusted comparison of baseline characteristics, smoking cessation assistance was more likely to be documented in the clinical encounters of older male patients, and for those with hypertension, dyslipidemia, diabetes mellitus, coronary artery disease, peripheral arterial disease, ischemic stroke/TIA, prior vascular intervention, heart failure, and atrial fibrillation or flutter (Table 1). Smoking cessation assistance was also more likely to be documented in encounters by physician provider as well in those generated from practices located in the Midwest, Northeast, or West Census regions and urban or suburban location. Considerable variation was noted in the provision of smoking cessation assistance across the 348 practices included in the present analysis.

**Figure 2:**
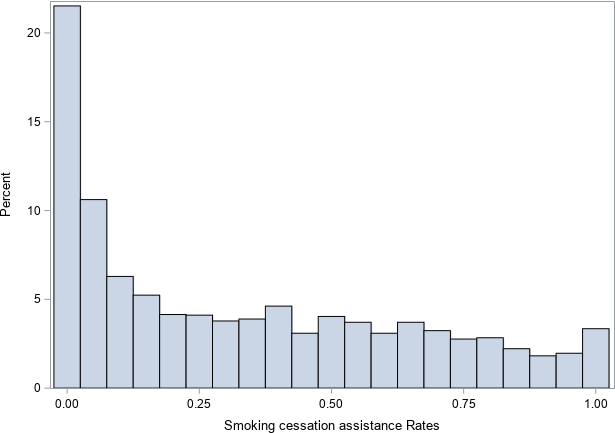
Histogram of smoking cessation assistance rates across providers in PINNACLE. The *x* axis represents percent of practices. The *y* axis represents assistance rates (0=assistance provided to no smokers, 0.5=assistance provided to 50% of smokers, 1=assistance provided to all smokers).

In a logistic regression model including the provider number as a random effect, there was significant variation in smoking cessation assistance among providers, as evidenced by MRR of 6.65 (95% CI, 6.37–7.01, Figure 2). With inclusion of the provider type and practice characteristics (Census region, urbanity) in the regression model, provider‐level variation persisted (MRR, 6.3; 95% CI 6.04–6.64). With inclusion of patient demographics and clinical variables in the regression model, MRR was slightly attenuated, but a high residual provider‐level variation in smoking cessation assistance remained (MRR, 6; 95% CI, 5.76–6.32).

Although the predictive variables in the final multivariable regression model accounted minimally for the large provider‐level variation, we observed significant association of various variables with smoking cessation assistance (Table 2). Female sex (odds ratio [OR], 1.18; 95% CI, 1.16–1.21), history of hypertension (OR, 1.28; 95% CI, 1.25–1.31), dyslipidemia (OR, 1.49; 95% CI, 1.45–1.52), coronary artery disease (OR, 1.28; 95% CI, 1.25–1.32), peripheral arterial disease (OR, 1.73; 95% CI, 1.68–1.78), TIA or ischemic stroke (OR, 1.14; 95% CI, 1.10–1.18), prior vascular intervention (OR, 1.04; 95% CI, 1.01–1.08), heart failure (OR, 1.06; 95% CI, 1.03–1.10) and practice location in Midwest region (OR, 1.61 versus West region; 95% CI, 1.18–2.21) were associated with higher odds of smoking cessation assistance documentation. On the contrary, older age (OR, 0.88 per 10‐year increase; 95% CI, 0.88–0.89), history of diabetes mellitus (OR, 0.84; 95% CI, 0.82–0.87), atrial fibrillation (OR, 0.93; 95% CI, 0.91–0.96), and practice location in South Census region (OR, 0.48 versus West region; 95% CI, 0.37–0.63) and rural (OR, 0.92 versus urban; 95% CI, 0.88–0.95) or suburban location (OR, 0.94 versus urban; 95% CI, 0.91–0.97) were inversely associated with smoking cessation assistance documentation. The association of diabetes mellitus and atrial fibrillation with lower rates of smoking cessation assistance were contrary to our hypothesis and could potentially be related to the inclusion of prevalent cardiovascular diseases strongly associated with smoking as a risk factor. We therefore performed exploratory analyses leveraging multivariable‐adjusted regression models where prevalent coronary artery disease, peripheral arterial disease, TIA/ischemic stroke, heart failure, and prior vascular interventions were not included in the model. The directionality of association of diabetes mellitus and atrial fibrillation with smoking cessation assistance in these models was similar to that observed in our original multivariable‐adjusted regression models (Table S2).

**Table 2:** Provider‐Level Variation and Association of Predictor Variables With Smoking Cessation Assistance

Variable	Model 1	Model 2		Model 3	
		Odds Ratio (95% CI)	*P* Value	Odds Ratio (95% CI)	*P* Value
Median rate ratio	6.65 (6.37–7.01)	6.30 (6.04–6.64)	–	6.00 (5.76–6.32)	–
Physician provider^a^		1.18 (0.93–1.49)	0.18	1.18 (0.94–1.49)	0.16
Northeast vs West region		1.06 (0.78–1.45)	0.71	1.07 (0.79–1.45)	0.37
Midwest vs West region		1.68 (1.21–2.32)	0.002	1.62 (1.18–2.22)	0.003
South vs West region		0.47 (0.36–0.61)	<0.001	0.48 (0.37–0.63)	<0.001
Rural vs urban location		0.92 (0.89–0.95)	<0.001	0.92 (0.88–0.95)	<0.001
Suburban vs urban location		0.94 (0.91–0.96)	<0.001	0.94 (0.91–0.97)	<0.001
Age (per 10‐year increase)				0.88 (0.88–0.89)	<0.001
Female sex				1.18 (1.16–1.21)	<0.001
Hypertension				1.28 (1.25–1.31)	<0.001
Diabetes mellitus				0.84 (0.82–0.87)	<0.001
Dyslipidemia				1.49 (1.45–1.52)	<0.001
Coronary artery disease				1.28 (1.25–1.32)	<0.001
Peripheral arterial disease				1.73 (1.68–1.78)	<0.001
TIA or ischemic stroke				1.14 (1.10–1.18)	<0.001
Heart failure				1.06 (1.03–1.10)	<0.001
Atrial fibrillation or flutter				0.93 (0.91–0.96)	<0.001
Prior vascular intervention				1.04 (1.01–1.08)	0.017

Model 1 (empty model) included provider number and was included as a random effect to estimate the variation in smoking cessation assistance among providers. Model 2 included practice‐level variables (practice number, Census region, and urbanity) and provider type (physician vs advanced practice providers) as fixed effect variables. Model 3 additionally included patient‐level variables (age, sex, hypertension, diabetes mellitus, dyslipidemia, coronary artery disease, peripheral arterial disease, TIA or cerebrovascular accident, heart failure, atrial fibrillation or flutter, and prior vascular intervention). TIA inidcates transient ischemic attack.

a Physician provider vs advanced practice provider.

## Discussion

In this cross‐sectional analysis of a large contemporary sample of smokers receiving outpatient care in US cardiology practices enrolled in the NCDR PINNACLE registry, we observed low rates of smoking cessation assistance documentation. We observed a large variation in the provision of smoking cessation assistance among providers, an observation that persisted after adjustment for measured provider, practice, and patient characteristics. Older age, diabetes mellitus, atrial fibrillation, practice location in the South Census region and rural or suburban areas were associated with lower odds of receiving smoking cessation assistance. Our findings suggest that a large deficit in the provision of smoking cessation assistance exists in the US ambulatory cardiology practices and that the commonly measured provider, practice, and patient characteristics account minimally for the large provider‐level variation in smoking cessation assistance. These findings call for immediate action from providers and public health organizations to improve the adherence to the provision of smoking cessation assistance in outpatient cardiology practices.

Cigarette smoking remains a common and modifiable risk factor for CVD and non‐CVDs.^1^, ^2^ Smoking cessation at any age is associated with a significant reduction in the risk of CVDs within first 5 years of cessation.^3^, ^4^, ^6^ In an analysis from the National Health Interview Survey conducted by the Centers for Disease Control and Prevention, nearly two thirds of current smokers intended to quit smoking and over half made attempts at quitting within the previous year.^5^ However, only a third of surveyed smokers reported receipt of counseling and/or medications when trying to quit. The findings made in our analyses are in line with the Centers for Disease Control and Prevention findings. Only 1 in 3 encounters for current smokers in the PINNACLE registry had documentation about the provision of smoking cessation assistance. Our findings suggest a significant deficit and opportunity for improvement, especially when one considers the fact that a large number of patients included in the PINNACLE registry were presenting for a cardiology visit because of a preexisting CVD. In prior analyses conducted from the National Ambulatory Medical Care Survey data (1994–1996 and 2001–2003), only 1 in 4 to 1 in 5 current smokers received cessation assistance in primary care and specialty clinics.^8^, ^9^. A more recent patient survey data from primary care provider and HIV clinics reported slightly better assistance rates (45%).^7^ Taken together, our findings suggest that a significant deficit in smoking cessation assistance exists, especially when one considers the fact that only 3.4% of current smokers (ie, 10% of encounters in which smoking cessation counseling was provided) received pharmacotherapy to assist with cessation in our analyses.

In a survey of nearly 150 cardiologists by the American College of Cardiology, the majority of participants reported that they assess the readiness to quit and advise the patients to quit smoking.^14^ However, just over two thirds felt comfortable with referral to the smoking cessation programs and only half felt comfortable prescribing pharmacotherapy to assist in smoking cessation. Seventy‐five percent of surveyed participants identified the desire and need to learn more about effective cessation therapies. In another recent European survey, Kotz et al^21^ surveyed 600 randomly selected Dutch cardiologists regarding their smoking cessation assistance patterns. Only a third of participants self‐reported providing smoking cessation counseling regularly, a remarkably similar rate to what was observed in our findings from the PINNACLE registry.

The ambulatory clinic visit represents a valuable opportunity for cardiology practitioners to emphasize smoking as an important, modifiable CVD risk factor and to provide guidance during a “teachable moment.” One of the unique findings from our analyses is that commonly measured provider, practice, and patient characteristics account minimally for the large provider‐level variation in smoking cessation assistance. The 2 aforementioned surveys suggest that one of the factors responsible for low rates of smoking cessation assistance observed in our study may include the fact that many providers do not feel comfortable with pharmacotherapies for smoking cessation, nor are they familiar with available counseling resources. Although additional data from US providers on this issue are lacking, in a recent survey of 371 French cardiologists, the lack of familiarity with smoking cessation guidelines, lack of adequate reimbursement for counseling, and, potentially, the physician's own smoking status were identified as the factors mediating variation in smoking cessation assistance.^13^ Furthermore, the lack of primary ownership in managing what are perceived as “nonprimary CVD” medical problems, might also play a role in creating confusion and the assumption that smoking cessation assistance is being provided by primary care physicians.^22^ Our findings provide actionable information for providers and public health organizations to address this concerning and largely idiosyncratic provider‐level deficit in smoking cessation assistance. Some of the potential methods that can be leveraged to address this deficit are provider training in smoking cessation counseling/motivational interviewing/pharmacotherapy, clinical workflow improvement (with assistance from paraprofessionals),^23^ patient education, provision of adequate reimbursement, and, potentially, provider incentive for smoking cessation assistance.^24^ Although we did not assess for the influence of health insurance type on the provision of smoking cessation assistance, smoking cessation counseling was a quality metric emphasized by Medicare (Physician Quality Reporting System) during our study period, and therefore penalties were imposed for nondocumentation of counseling for certain patients included in our sample.^25^ Further research is needed to determine whether reimbursement payments/penalties for providing smoking cessation assistance are exerting a positive effect on practice patterns.

In addition to the large deficit and provider‐level variation, we observed regional and rural disparities in smoking cessation assistance. Practice location in the South Census region (versus the West region) and rural/suburban locations (versus urban location) had an inverse association with smoking cessation assistance. These disparities are concerning considering the higher burden of CVD and CVD risk factors (including smoking) in the South region and rural America.^26^ Interestingly, provider type (physician versus advanced practice provider) was not associated with smoking cessation assistance. This finding supports the American College of Cardiology's recommendation regarding the training and incorporation of advanced practice providers in the cardiovascular team–based model of healthcare delivery to address the critical workforce shortage.^27^ We also identified age‐related disparities in the provision of smoking cessation assistance despite the proven benefit of smoking cessation across all age groups in prior studies.^28^

Similarly, the patients with diabetes mellitus and atrial fibrillation were less likely to receive smoking cessation assistance. Smoking has been associated with poor glycemic control and increased risk for complications including CVD in patients with diabetes mellitus.^29^, ^30^, ^31^ The inverse association of smoking cessation assistance with diabetes mellitus observed in our analyses might reflect a knowledge gap in practitioners about the causal association of smoking with diabetes mellitus and/or concern about worsening glycemic control with smoking cessation.^32^ The prognostic importance of cigarette smoking as a risk factor for atrial fibrillation has been recognized only in recent population‐based studies.^33^, ^34^, ^35^ The inverse association of atrial fibrillation with smoking cessation assistance in our analyses likely limited understanding in providers about the association of smoking and atrial fibrillation. These findings provide important opportunities to address the knowledge gap among providers with further educational efforts.

### Strengths and Limitations

Our findings should be interpreted in context of the strengths and limitations of the study. We used the detailed clinical data collected from representative nationwide practices enrolled in the PINNACLE registry. Our large sample size adds confidence to our findings. However, we acknowledge several limitations. First, ascertainment of smoking cessation assistance relied on either documentation of smoking cessation counseling or prescription of smoking cessation pharmacotherapies. It is plausible that in certain clinic encounters, counseling may have been provided but not documented in the clinic note. However, it should be noted that the rates of smoking cessation assistance in our study were comparable to those reported by surveyed US cardiologists. Second, site participation in the PINNACLE registry is voluntary. The practice selection bias inherent in PINNACLE means that the rates of smoking cessation assistance are likely even lower in the non‐PINNACLE practices. Third, we did not obtain data on smoking cessation rates and efficacy of smoking cessation assistance. Fourth, we did not adjust for social determinants of health (race, income, education), which might affect the rates of smoking cessation assistance. Fifth, regional differences in health funding and policies might mediate some of the variation in smoking cessation assistance patterns. Unfortunately, these data were not collected in the PINNACLE registry. However, we adjusted for random effect from the practices in all our multivariable‐models, which likely adjusted for all practice‐level effects, measured and unmeasured. Sixth, the PINNACLE electronic medical record mapping algorithm may not identify referrals to the smoking cessation programs, such 1‐800‐QUITNOW. But the algorithm accurately captures the cessation counseling that accompanies these referrals. Therefore, we do not anticipate that the algorithm significantly underestimated the rates of smoking cessation assistance.

## Conclusions

In this large cross‐sectional analysis of a US ambulatory cardiology registry with periodically audited high‐quality data and representative sites across the United States, we identified low rates of smoking cessation assistance provided to current smokers presenting for an outpatient visit. These findings call to action and educate providers in the provision of smoking cessation assistance. We identified both patient‐ and practice‐level predictors of receiving smoking cessation assistance. However, despite adjusting for various predictors, a large provider‐level variation in smoking cessation assistance persisted. Our findings form the basis for further investigation into other contributors to smoking cessation assistance, such as economic, social, environmental, and organizational factors, that could enhance the rates of smoking cessation assistance and reduce variation in care.

## Sources of Funding

This study was funded by the American College of Cardiology's National Cardiovascular Data Registry.

## Disclosures

Dr McManus is supported by 1U01HL105268‐01, KL2RR031981, R01HL126911, and R01HL137794 from the National Heart, Lung, and Blood Institute of the National Institutes of Health, HRS174612 from the Heart Rhythm Society, and Grant 1522052 from the National Science Foundation. Dr McManus also receives research support from Bristol‐Myers Squibb, Pfizer, Biotronik, and Philips Healthcare, and has consulted for Bristol‐Myers Squibb, FlexCon, Samsung, Philips, and Pfizer. Dr McManus has equity in Mobile Sense Technologies, LLC. The remaining authors have no disclosures to report.

## Supporting information


**Table S1.** Baseline Patient‐, Provider‐, and Practice‐Level Characteristics of Patients Stratified by Receipt of Smoking Cessation Pharmacotherapy
**Table S2.** Association of Diabetes Mellitus and Atrial Fibrillation with Smoking Cessation Assistance
