# Supplementary material for: Provider‐Level Variation in Smoking Cessation Assistance Provided in the Cardiology Clinics: Insights From the NCDR PINNACLE Registry
Source: J Am Heart Assoc. 2019 Jun 28;8(13):e011412. doi: 10.1161/JAHA.118.011307 (PMC6662347; doi:10.1161/JAHA.118.011307)
Supplement: Supplementary file 1 — Table S1. Baseline Patient‐, Provider‐, and Practice‐Level Characteristics of Patients Stratified by Receipt of Smoking Cessation Pharmacotherapy Table S2. Association of Diabetes Mellitus and Atrial Fibrillation with Smoking Cessation Assistance [file JAH3-8-e011412b-s001.pdf]

SUPPLEMENTAL MATERIAL

Table S1. Baseline Patient, Provider and Practice-level Characteristics of Patients Stratified by Receipt of Smoking Cessation Pharmacotherapy.

Variable, n(%) unless specified	Smoking Cessation Pharmacotherapy Yes N=11223	No N=101661	Standardized Difference (%)
**Patient-level characteristics**			
Age, years, mean±SD	56±13	59±14	19
Women	6335 (56%)	43731 (43%)	27
Hypertension	7079 (63%)	72349 (71%)	17
Diabetes	2665 (24%)	20854 (21%)	8
Dyslipidemia	7030 (63%)	66622 (66%)	6
Coronary artery disease	4403 (39%)	47173 (46%)	15
Peripheral arterial disease	1987 (18%)	19627 (19%)	4
TIA or ischemic stroke	1194 (11%)	9430 (9%)	5
Heart failure	1500 (13%)	17366 (17%)	10
Atrial fibrillation or flutter	1232 (11%)	15926 (16%)	14
Prior vascular intervention	1683 (15%)	16567 (16%)	4
**Provider & Practice-level characteristics**			
Physician provider	10586 (94%)	95227 (94%)	2
US Census region			N/A
Northeast region	1631 (15%)	17442 (17%)	
Midwest region	1862 (17%)	19633 (19%)	
South region	6808 (61%)	55069 (54%)	
West region	922 (8%)	9517 (9%)	
Urbanity			N/A
Rural	4481 (42%)	40389 (41%)	
Suburban	4325 (41%)	38796 (39%)	
Urban	1916 (18%)	20577 (21%)	

Only participants who received smoking cessation counseling are included here. N/A= not applicable.

Table S2. Association of Diabetes and Atrial Fibrillation with Smoking Cessation Assistance.

Variable	Odds Ratio (95% CI)	P value
Median rate ratio	6.12 (5.87 – 6.45)	--
Physician provider*	1.19 (0.94 – 1.50)	0.15
Northeast vs. West region	1.05 (0.77 – 1.43)	0.36
Midwest vs. West region	1.66 (1.21 – 2.28)	0.002
South vs. West region	0.47 (0.36 – 0.61)	<0.001
Rural vs. urban location	0.92 (0.89 – 0.95)	<0.001
Suburban vs. urban location	0.94 (0.91 – 0.97)	<0.001
Age (per 10 year increase)	0.91 (0.91 – 0.97)	<0.001
Female sex	1.15 (1.12 – 1.17)	<0.001
Hypertension	1.32 (1.29 – 1.35)	<0.001
Diabetes	0.91 (0.89 – 0.94)	<0.001
Dyslipidemia	1.64 (1.61 – 1.68)	<0.001
Atrial fibrillation or flutter	0.93 (0.91 – 0.96)	<0.001

*Physician provider vs. advanced practice provider.
